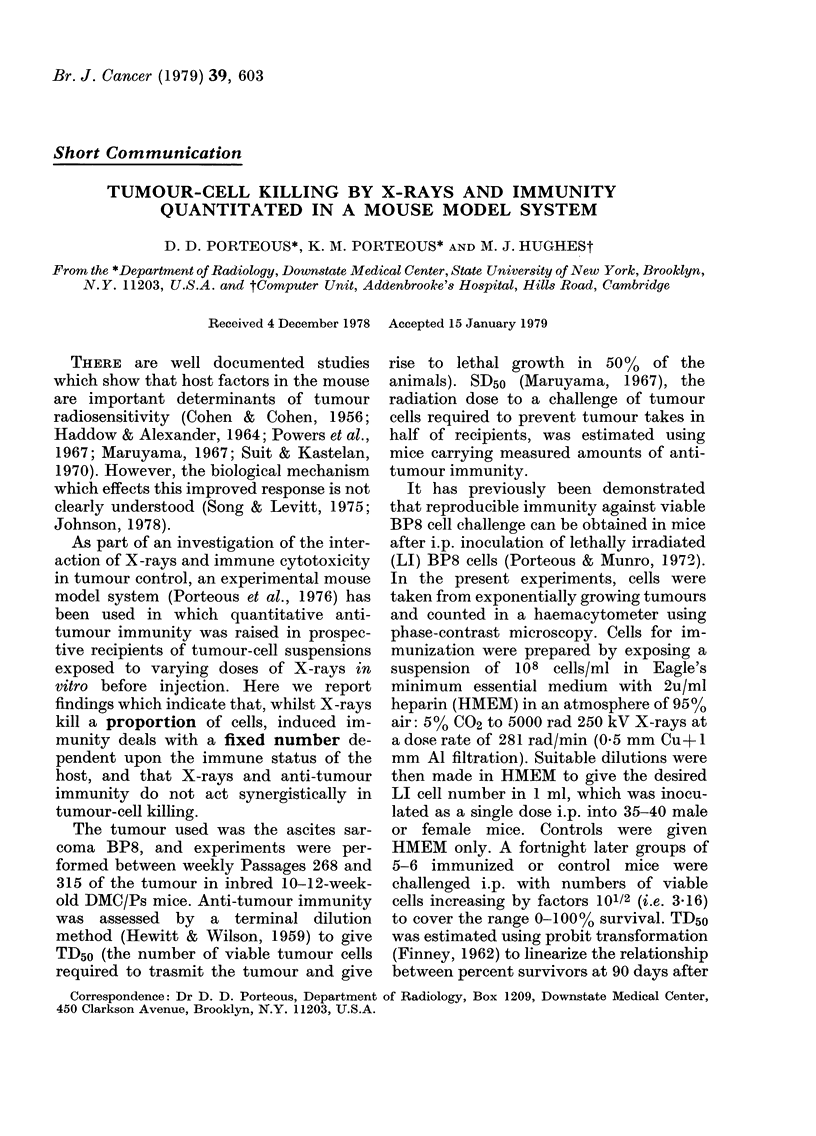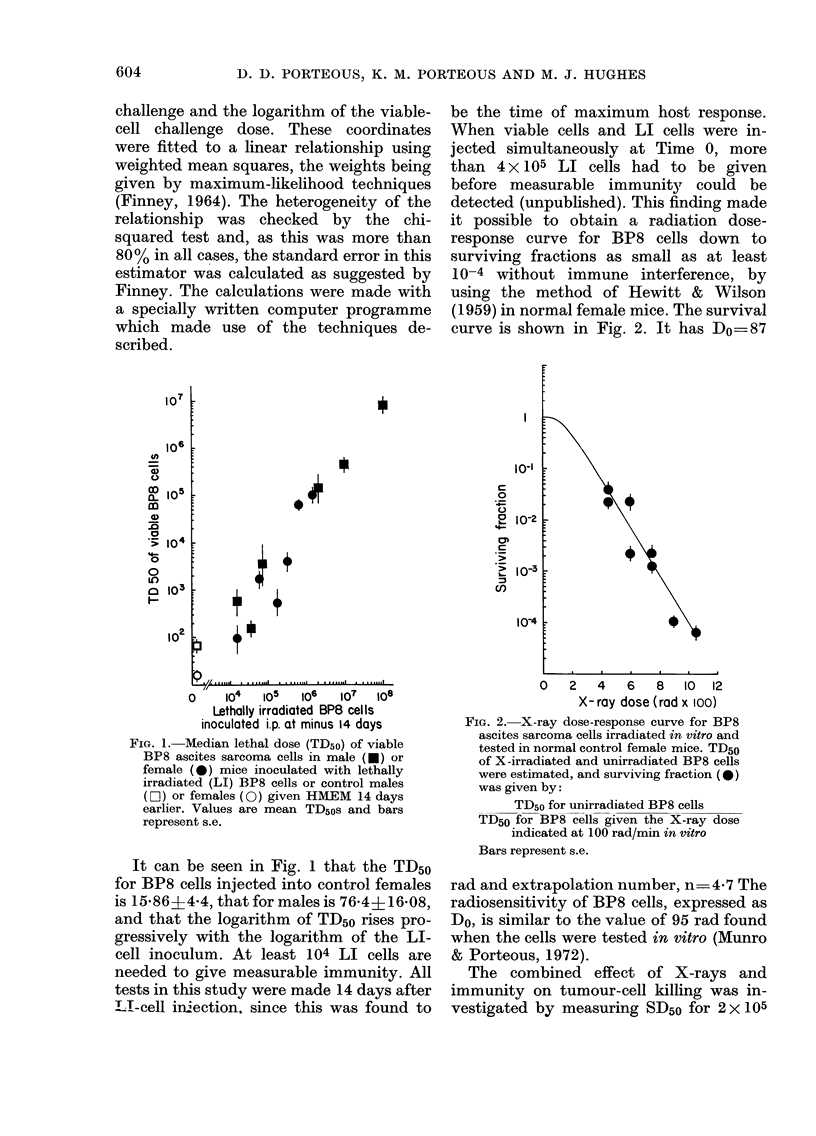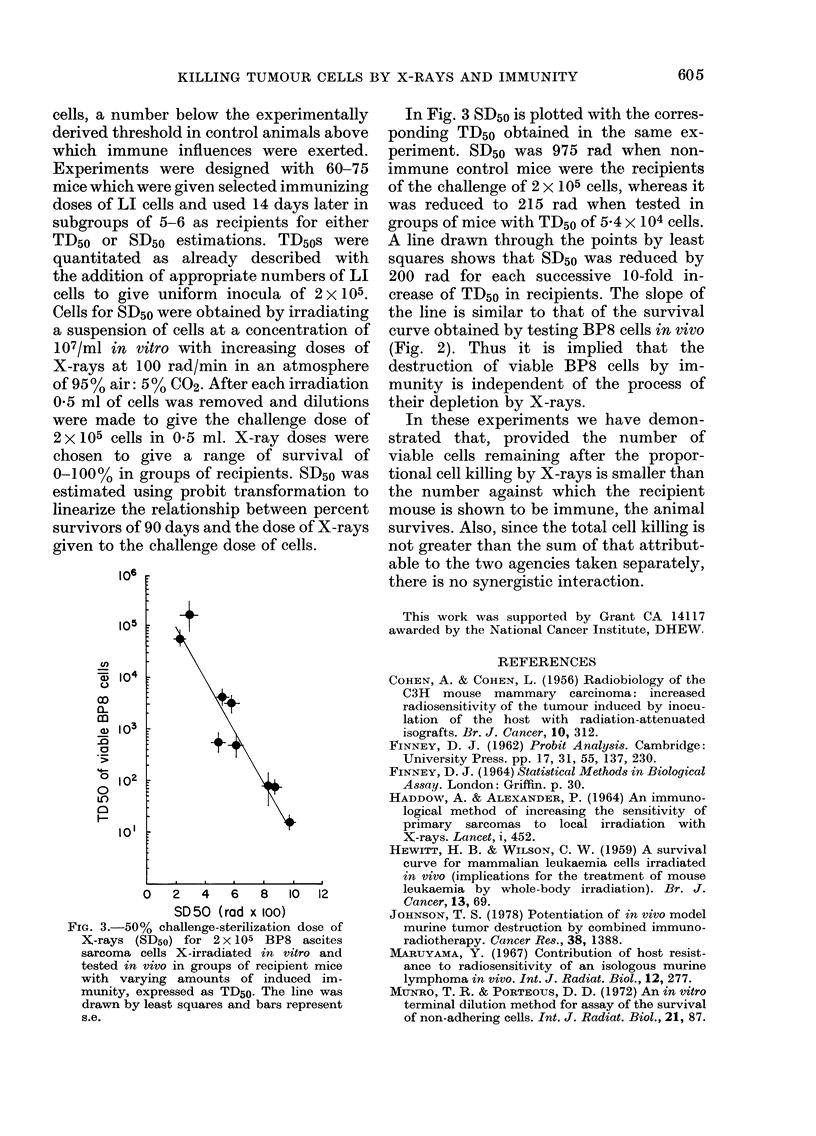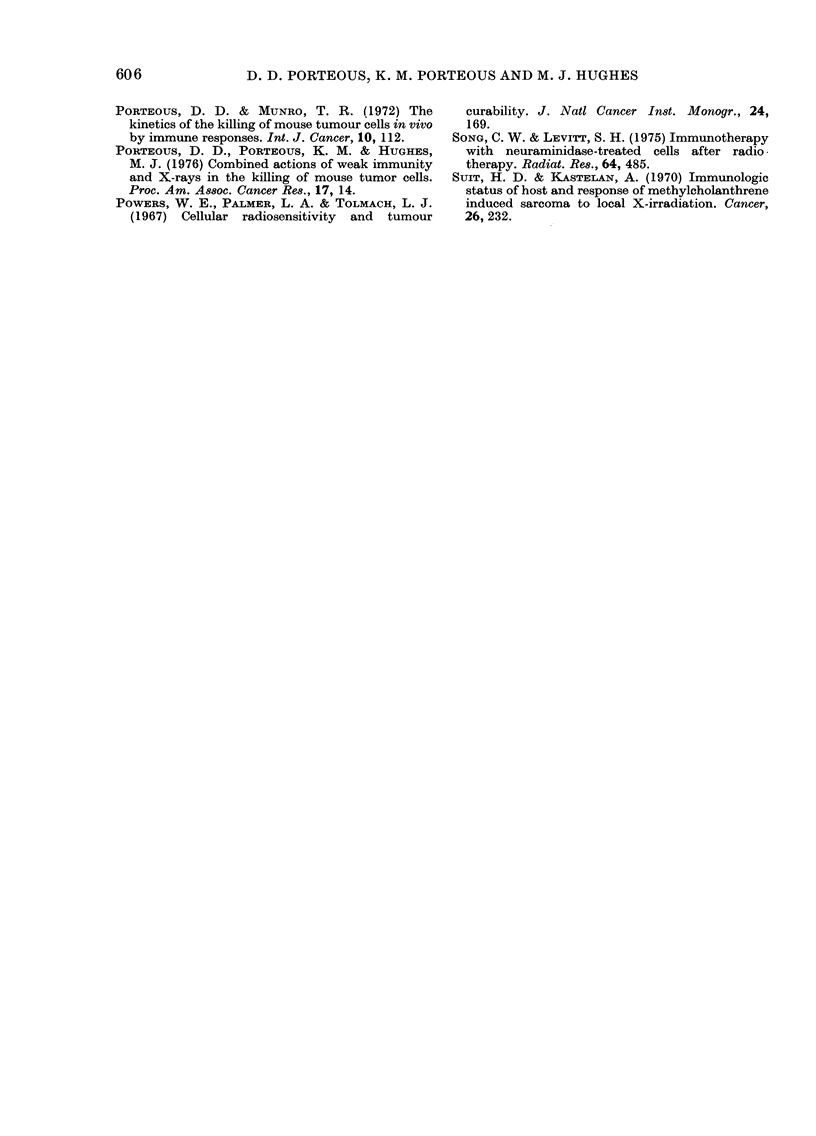# Tumour-cell killing by X-rays and immunity quantitated in a mouse model system.

**DOI:** 10.1038/bjc.1979.110

**Published:** 1979-05

**Authors:** D. D. Porteous, K. M. Porteous, M. J. Hughes


*Br. J. Cancer* (1979) **39**, 603

***Short Communication***

### TUMOUR-CELL KILLING BY X-RAYS AND IMMUNITY QUANTITATED IN A MOUSE MODEL SYSTEM

D. D. PORTEOUS*, K. M. PORTEOUS* AND M. J. HUGHES†

*From the *Department of Radiology, Downstate Medical Center, State University of New York, Brooklyn, N.Y.* 11203, *U.S.A. and †Computer Unit, Addenbrooke's Hospital, Hills Road, Cambridge*

Received 4 December 1978 Accepted 15 January 1979

THERE are well documented studies which show that host factors in the mouse are important determinants of tumour radiosensitivity (Cohen & Cohen, 1956; Haddow & Alexander, 1964; Powers *et al.*, 1967; Maruyama, 1967; Suit & Kastelan, 1970). However, the biological mechanism which effects this improved response is not clearly understood (Song & Levitt, 1975; Johnson, 1978).

As part of an investigation of the interaction of X-rays and immune cytotoxicity in tumour control, an experimental mouse model system (Porteous *et al.*, 1976) has been used in which quantitative anti-tumour immunity was raised in prospective recipients of tumour-cell suspensions exposed to varying doses of X-rays *in vitro* before injection. Here we report findings which indicate that, whilst X-rays kill a **proportion** of cells, induced immunity deals with a **fixed number** dependent upon the immune status of the host, and that X-rays and anti-tumour immunity do not act synergistically in tumour-cell killing.

The tumour used was the ascites sarcoma BP8, and experiments were performed between weekly Passages 268 and 315 of the tumour in inbred 10–12-week-old DMC/Ps mice. Anti-tumour immunity was assessed by a terminal dilution method (Hewitt & Wilson, 1959) to give $TD_{50}$ (the number of viable tumour cells required to trasmit the tumour and give rise to lethal growth in 50% of the animals). $SD_{50}$ (Maruyama, 1967), the radiation dose to a challenge of tumour cells required to prevent tumour takes in half of recipients, was estimated using mice carrying measured amounts of anti-tumour immunity.

It has previously been demonstrated that reproducible immunity against viable BP8 cell challenge can be obtained in mice after i.p. inoculation of lethally irradiated (LI) BP8 cells (Porteous & Munro, 1972). In the present experiments, cells were taken from exponentially growing tumours and counted in a haemacytometer using phase-contrast microscopy. Cells for immunization were prepared by exposing a suspension of $10^8$ cells/ml in Eagle's minimum essential medium with 2u/ml heparin (HMEM) in an atmosphere of 95% air: 5% $CO_2$ to 5000 rad 250 kV X-rays at a dose rate of 281 rad/min (0·5 mm Cu+1 mm Al filtration). Suitable dilutions were then made in HMEM to give the desired LI cell number in 1 ml, which was inoculated as a single dose i.p. into 35–40 male or female mice. Controls were given HMEM only. A fortnight later groups of 5–6 immunized or control mice were challenged i.p. with numbers of viable cells increasing by factors $10^{1/2}$ (*i.e.* 3·16) to cover the range 0–100% survival. $TD_{50}$ was estimated using probit transformation (Finney, 1962) to linearize the relationship between percent survivors at 90 days after

Correspondence: Dr D. D. Porteous, Department of Radiology, Box 1209, Downstate Medical Center, 450 Clarkson Avenue, Brooklyn, N.Y. 11203, U.S.A.

challenge and the logarithm of the viable-cell challenge dose. These coordinates were fitted to a linear relationship using weighted mean squares, the weights being given by maximum-likelihood techniques (Finney, 1964). The heterogeneity of the relationship was checked by the chi-squared test and, as this was more than 80% in all cases, the standard error in this estimator was calculated as suggested by Finney. The calculations were made with a specially written computer programme which made use of the techniques described.

be the time of maximum host response. When viable cells and LI cells were injected simultaneously at Time 0, more than $4 \times 10^5$ LI cells had to be given before measurable immunity could be detected (unpublished). This finding made it possible to obtain a radiation dose-response curve for BP8 cells down to surviving fractions as small as at least $10^{-4}$ without immune interference, by using the method of Hewitt & Wilson (1959) in normal female mice. The survival curve is shown in Fig. 2. It has $D_0 = 87$

FIG. 1.—Median lethal dose ($TD_{50}$) of viable BP8 ascites sarcoma cells in male (■) or female (●) mice inoculated with lethally irradiated (LI) BP8 cells or control males (□) or females (○) given HMEM 14 days earlier. Values are mean $TD_{50}$s and bars represent s.e.

FIG. 2.—X-ray dose-response curve for BP8 ascites sarcoma cells irradiated *in vitro* and tested in normal control female mice. $TD_{50}$ of X-irradiated and unirradiated BP8 cells were estimated, and surviving fraction (●) was given by:

$$\frac{TD_{50} \text{ for unirradiated BP8 cells}}{TD_{50} \text{ for BP8 cells given the X-ray dose indicated at 100 rad/min } \textit{in vitro}}$$

Bars represent s.e.

It can be seen in Fig. 1 that the $TD_{50}$ for BP8 cells injected into control females is $15{\cdot}86 \pm 4{\cdot}4$, that for males is $76{\cdot}4 \pm 16{\cdot}08$, and that the logarithm of $TD_{50}$ rises progressively with the logarithm of the LI-cell inoculum. At least $10^4$ LI cells are needed to give measurable immunity. All tests in this study were made 14 days after LI-cell injection, since this was found to rad and extrapolation number, $n = 4{\cdot}7$ The radiosensitivity of BP8 cells, expressed as $D_0$, is similar to the value of 95 rad found when the cells were tested *in vitro* (Munro & Porteous, 1972).

The combined effect of X-rays and immunity on tumour-cell killing was investigated by measuring $SD_{50}$ for $2 \times 10^5$

cells, a number below the experimentally derived threshold in control animals above which immune influences were exerted. Experiments were designed with 60–75 mice which were given selected immunizing doses of LI cells and used 14 days later in subgroups of 5–6 as recipients for either $TD_{50}$ or $SD_{50}$ estimations. $TD_{50}$s were quantitated as already described with the addition of appropriate numbers of LI cells to give uniform inocula of $2 \times 10^5$. Cells for $SD_{50}$ were obtained by irradiating a suspension of cells at a concentration of $10^7$/ml *in vitro* with increasing doses of X-rays at 100 rad/min in an atmosphere of 95% air: 5% $CO_2$. After each irradiation 0·5 ml of cells was removed and dilutions were made to give the challenge dose of $2 \times 10^5$ cells in 0·5 ml. X-ray doses were chosen to give a range of survival of 0–100% in groups of recipients. $SD_{50}$ was estimated using probit transformation to linearize the relationship between percent survivors of 90 days and the dose of X-rays given to the challenge dose of cells.

FIG. 3.—50% challenge-sterilization dose of X-rays ($SD_{50}$) for $2 \times 10^5$ BP8 ascites sarcoma cells X-irradiated *in vitro* and tested *in vivo* in groups of recipient mice with varying amounts of induced immunity, expressed as $TD_{50}$. The line was drawn by least squares and bars represent s.e.

In Fig. 3 $SD_{50}$ is plotted with the corresponding $TD_{50}$ obtained in the same experiment. $SD_{50}$ was 975 rad when non-immune control mice were the recipients of the challenge of $2 \times 10^5$ cells, whereas it was reduced to 215 rad when tested in groups of mice with $TD_{50}$ of $5{\cdot}4 \times 10^4$ cells. A line drawn through the points by least squares shows that $SD_{50}$ was reduced by 200 rad for each successive 10-fold increase of $TD_{50}$ in recipients. The slope of the line is similar to that of the survival curve obtained by testing BP8 cells *in vivo* (Fig. 2). Thus it is implied that the destruction of viable BP8 cells by immunity is independent of the process of their depletion by X-rays.

In these experiments we have demonstrated that, provided the number of viable cells remaining after the proportional cell killing by X-rays is smaller than the number against which the recipient mouse is shown to be immune, the animal survives. Also, since the total cell killing is not greater than the sum of that attributable to the two agencies taken separately, there is no synergistic interaction.

This work was supported by Grant CA 14117 awarded by the National Cancer Institute, DHEW.

### REFERENCES

COHEN, A. & COHEN, L. (1956) Radiobiology of the C3H mouse mammary carcinoma: increased radiosensitivity of the tumour induced by inoculation of the host with radiation-attenuated isografts. *Br. J. Cancer*, **10**, 312.

FINNEY, D. J. (1962) *Probit Analysis*. Cambridge: University Press. pp. 17, 31, 55, 137, 230.

FINNEY, D. J. (1964) *Statistical Methods in Biological Assay*. London: Griffin. p. 30.

HADDOW, A. & ALEXANDER, P. (1964) An immunological method of increasing the sensitivity of primary sarcomas to local irradiation with X-rays. *Lancet*, i, 452.

HEWITT, H. B. & WILSON, C. W. (1959) A survival curve for mammalian leukaemia cells irradiated *in vivo* (implications for the treatment of mouse leukaemia by whole-body irradiation). *Br. J. Cancer*, **13**, 69.

JOHNSON, T. S. (1978) Potentiation of *in vivo* model murine tumor destruction by combined immunoradiotherapy. *Cancer Res.*, **38**, 1388.

MARUYAMA, Y. (1967) Contribution of host resistance to radiosensitivity of an isologous murine lymphoma *in vivo*. *Int. J. Radiat. Biol.*, **12**, 277.

MUNRO, T. R. & PORTEOUS, D. D. (1972) An *in vitro* terminal dilution method for assay of the survival of non-adhering cells. *Int. J. Radiat. Biol.*, **21**, 87.

PORTEOUS, D. D. & MUNRO, T. R. (1972) The kinetics of the killing of mouse tumour cells *in vivo* by immune responses. *Int. J. Cancer*, **10**, 112.

PORTEOUS, D. D., PORTEOUS, K. M. & HUGHES, M. J. (1976) Combined actions of weak immunity and X-rays in the killing of mouse tumor cells. *Proc. Am. Assoc. Cancer Res.*, **17**, 14.

POWERS, W. E., PALMER, L. A. & TOLMACH, L. J. (1967) Cellular radiosensitivity and tumour curability. *J. Natl Cancer Inst. Monogr.*, **24**, 169.

SONG, C. W. & LEVITT, S. H. (1975) Immunotherapy with neuraminidase-treated cells after radiotherapy. *Radiat. Res.*, **64**, 485.

SUIT, H. D. & KASTELAN, A. (1970) Immunologic status of host and response of methylcholanthrene induced sarcoma to local X-irradiation. *Cancer*, **26**, 232.